# Implementation of a Vector-based Tracking Loop Receiver in a Pseudolite Navigation System

**DOI:** 10.3390/s100706324

**Published:** 2010-06-25

**Authors:** Hyoungmin So, Taikjin Lee, Sanghoon Jeon, Chongwon Kim, Changdon Kee, Taehee Kim, Sanguk Lee

**Affiliations:** 1 School of Mechanical and Aerospace Engineering and SNU-IAMD, Seoul National University, Daehak-dong, Kwanak-gu, Seoul, 151–744, Korea; E-Mails: hyoungmin.so@gmail.com (H.S.) taikjin@snu.ac.kr (T.L.); nan772@snu.ac.kr (C.K.); kee@snu.ac.kr (C.K.); 2 Satellite Control & Navigation Team, Electronics and Telecommunications Research Institute, 138 Gajeongno, Yuseong-gu, Daejeon, 305–700, Korea; E-Mails: thkim72@etri.re.kr (T.K.); slee@etri.re.kr (S.L.)

**Keywords:** GPS, software receiver, vector tracking loop, pseudolite, indoor navigation

## Abstract

We propose a vector tracking loop (VTL) algorithm for an asynchronous pseudolite navigation system. It was implemented in a software receiver and experiments in an indoor navigation system were conducted. Test results show that the VTL successfully tracks signals against the near–far problem, one of the major limitations in pseudolite navigation systems, and could improve positioning availability by extending pseudolite navigation coverage.

## 1. Introduction

Recently, there has been much research on the Global Navigation Satellite System (GNSS) and its applications are spread over many areas. Location-based services are a good example of GNSS applications. As location-based services and applications spread, the need for positioning availability is increasing, even in places where positioning using the GNSS has not been available, such as indoors, urban canyons and other GNSS shadow areas. There have been many studies to increase GNSS positioning availability. A pseudolite (PL) is one of the powerful candidates [1]. A PL is a transmitter broadcasting a GNSS-like signal that can enable conventional GNSS users to do positioning. By installing PLs indoors or other places where the GNSS signal is not available, users can navigate seamlessly with a conventional GNSS receiver. To improve GNSS navigation availability, PLs are very useful because the conventional GNSS user is not required to change receiver hardware.

In this paper, meter-level PL navigation utilizing pseudorange measurements is a main consideration. There could be many applications. For indoor navigation, a meter-level PL is applicable to large-scale area where a GNSS receiver-equipped vehicle and equipment could be operated. A large-scale warehouse of a logistics center, an indoor shipbuilding plant, an indoor or underground parking lot of a big supermarket, an indoor amusement park, a large and long tunnel are good examples. Indoor PL navigation system has an advantage over the other indoor systems in that indoor/outdoor seamless navigation with only one user equipment could be achievable. Outdoor use of PLs is useful to cooperate with GNSS to improve navigation availability where the visibility of GNSS satellites is not fully obtained. It could be applicable to urban canyons or mountain valley regions. Maritime navigation is a good application of meter-level accuracy system. PLs could increase integrity of ship docking, harbor loading and unloading applications. PLs also could be an independent navigation system as a backup for wartime. PLs could enable the consistent use of GNSS-related equipment.

There are, however, still some limitations in managing PL navigation systems [2]. The near–far problem is one of the most critical limitations, especially for indoor PL navigation. The near–far problem refers to the variation in received signal strength with respect to the relative distance between the user and a PL. Because PLs are installed in a small area relative to GNSS satellites, the distances between the user and the PLs change rapidly compared with GNSS navigation. When the user approaches a specific PL, the received signal power from it is much higher than that from the others and it saturates the automatic gain control (AGC) of the receiver’s RF front end. This AGC saturation causes signal to noise ratio degradation of more distant PL signals. Because of this, PL navigation coverage is restricted to regions that are almost equidistant from all the PLs. Even when all the PLs are in appropriate locations, tuning the transmitting power of each PL remains a problem, and is very troublesome in indoor navigation [3].

There are some techniques to mitigate the near–far problem. A pulsing scheme is one of the most popular solutions [4]. Each PL signal transmission is turned on and off in fixed duty cycles so that they do not interfere with each other. While some near PLs saturate the user’s AGC during their own duty cycles, the other PL signals can be received during their own duty cycles. Pulsing is effective but cannot be a complete solution, because its performance decreases as the number of signal sources increases. In addition, fine scheduling on the pulsing timing according to the PL constellation and AGC characteristics is required.

While pulsing is a solution for the near–far problem in PL systems, we propose a vector tracking loop (VTL) algorithm with PL systems as a new solution, to be implemented in a receiver. Combining a pulsing scheme in PLs and a VTL in a receiver could make a more robust PL navigation system and improve navigation availability.

The main feature of the VTL is that it has one big loop that combines tracking and navigation. Conventional receivers use an independent tracking loop (ITL) and navigation functions. VTL tracking control input is generated from pseudorange and range-rate estimates that are estimated from navigation results, while the navigation results are calculated from the tracking results. Tracking results, *i.e.*, discriminator output, are not directly connected to the tracking control inputs, but are used in estimating pseudorange and range rate from receiver position, velocity and satellite position. These in turn are used to generate tracking control inputs. In this case, even though some satellite signals are attenuated or blocked at times, the receiver can track them using the navigation results obtained from undisturbed visible satellites. It can make use of the redundancy of visible satellites. This is a well-known technique for improving tracking robustness. Spilker [1] commented that in the nonlinear conditions in PL navigation systems, a VTL should improve tracking performance. However, the application of VTL to PL systems has not yet been properly studied. We propose a VTL algorithm applicable to asynchronous PL navigation systems, and assess its ability to mitigate the near–far problem and improve PL navigation availability.

This paper starts with a brief review of the VTL concept, comparing it with a conventional ITL in Section 2. Section 3 describes the construction of a vector delay/frequency lock loop (VDFLL) based on the extended Kalman filter (EKF). In Section 4, a measurement model and a navigation algorithm for asynchronous PL navigation systems are reviewed. Section 5 proposes a VTL algorithm for an asynchronous PL navigation system and its receiver implementation. In Sections 6 and 7, a simulation and test results will be described. The test was performed using the Seoul National University GNSS Laboratory (SNUGL) indoor navigation system. The results show that VTL could be a good solution for the near–far problem and improve PL navigation availability.

## 2. Brief Review of VTL

In 1980, the basic concept of VTL was described in Copps’ paper [5]. It introduced a GPS/Inertial Navigation System (INS) composite filter utilizing the user’s navigation state to estimate tracking control input. Sennott proposed a combined tracker–navigator construction and presented simulation results showing improved tracking performance of an attenuated signal [6]. The term Vector Tracking Loop was first used by Spilker [7]. He proposed a vector delay-lock loop (VDLL) algorithm, combining all the tracking channels and navigation function. Implementation of EKF-based VDLL, mathematical derivation of noise performance improvement and several potential VTL advantages were addressed. A recent performance analysis was well described by Benson [8]. As software receivers developed, many studies of VTL were conducted [9]. Several VTL implementation methods in software receivers and their field test results showing improved tracking performance were reported in [10,11].

**Figure 1.**
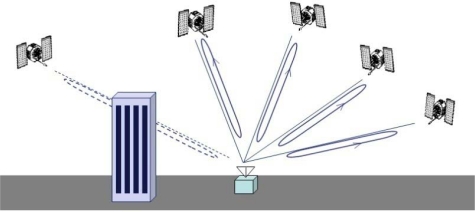
Independent tracking loop.

**Figure 2.**
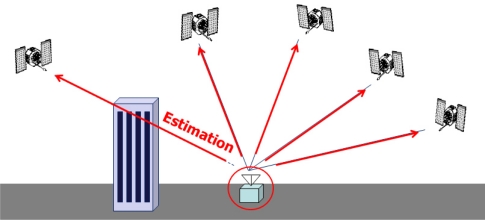
Vector tracking loop.

GPS/INS ultratight integration (deep coupling) was thoroughly investigated as a VTL application [12,13]. Recent VTL implementations with a variety of filters were analyzed and compared by Petovello [14]. Expansion of VTL to vector-based phase lock loop (VPLL) is an ongoing research theme in this field [15,16]. The concept of VTL is easily explained by comparing a conventional tracking loop and VTL, as shown in Figures 1 and 2, respectively. Figure 1 shows a conventional tracking loop composed of several ITLs tracking each satellite signal. Channel tracking results are transferred to the navigation function. All the channels are independent, and tracking and navigation functions are also independent. Figure 2 shows the VTL concept. Comparing with Figure 1, the discriminator outputs are directly connected to the tracking control input, while all the input is estimated from the user navigation result. Because the navigation result is derived from all channel tracking results, all the channels and navigation function are combined. This structure can track temporarily attenuated or blocked satellite signals because the navigation result can be derived from other visible satellites.

**Figure 3.**
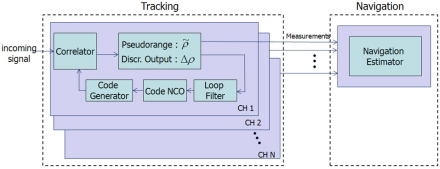

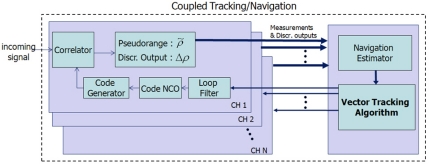
Receiver implementation of ITL (top) and VTL (bottom).

As a more concrete example, VTL and ITL construction in a GNSS receiver is shown in Figure 3. The top part of Figure 3 shows an ITL implementation.

Each channel’s tracking measurements are transferred to the navigation module. The measurements are generated in each channel tracking loop independently and the navigation module works separately. VTL, however, does not contain channel-independent tracking loops. Instead, the navigation module collects all the channel tracking results and estimates each tracking control input. Thus, there is one big loop including tracking channels and navigation module.

## 3. VTL Implementation

There have been many studies of VTL implementation. The initial VDLL implementation concept was well explained in [7]. Recently, [13,17] described VDLL, VFLL and VDFLL with EKF. The VTL implementation in this paper does not significantly differ from the previous studies. We implemented VDFLL with EKF in a software receiver.

**Figure 4.**
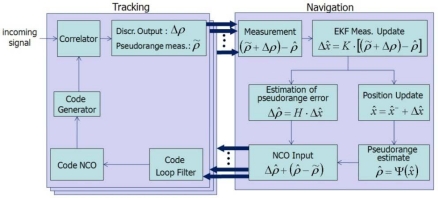
Construction of VDLL.

Figure 4 shows the workflow of the VDLL we implemented. Its notation follows [13]. The VTL algorithm starts with the discriminator output Δ*ρ̃* and the pseudorange measurement *ρ̃*:

*ρ̃*: Measured pseudorange
Δ*ρ̃*: Discriminator output (Δ*ρ̃* = *ρ_true_* − *ρ̃* + *n*)
*n* : pseudorange estimation noise
*ρ_true;_*: true pseudorange (unknown)
*ρ̂*: pseudorange estimate (*ρ̂* = Ψ(*x̂*) = |*x_sat_* − *x̂*| + *c* · *t_u_*)
*x̂* : user position state vector
*x_sat_* : satellite position vector
*c* : speed of light
*t_u_* : user receiver clock offset
*K* : Kalman gain
*H* : Geometric matrix consisted of line of sight vectors.

In the discriminator output model, Δ*ρ̃* ≈ *ρ_true_ − ρ̃* + *n*; the true pseudorange *ρ_true_* is unknown. It is therefore approximated as:

(1) $${\hat{\rho }}_{\mathrm{true}}\approx \Delta \tilde{\rho }+\tilde{\rho }$$

**Figure 5.**
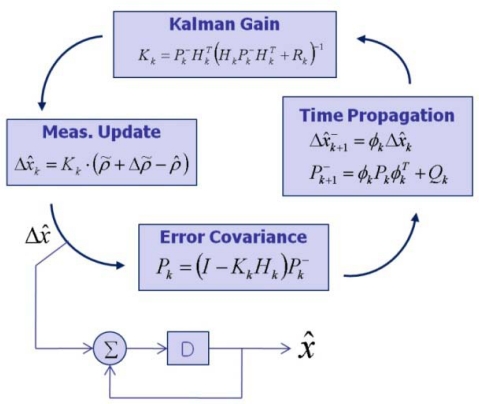
EKF construction.

Next, an error estimate (*ρ̃* + Δ*ρ*)− *ρ̂* can be used as a Kalman filter input. The construction of the EKF is established, as shown in Figure 5 [18].

From the output of the navigation EKF, navigation states are updated. From these results, updated pseudorange estimates are calculated:

(2) $$\hat{\rho }=\Psi \left(\hat{x}\right)=\left|x_{\mathrm{sat}}-\hat{x}\right|+c\cdot t_{u}=\sqrt{{\left(x_{i}-x_{u}\right)}^{2}+{\left(y_{i}-y_{u}\right)}^{2}+{\left(z_{i}-z_{u}\right)}^{2}}+c\cdot t_{u},$$

where : *x_i_*, *y_i_*, *z_i_* : *x,y,z* position of *i*th satellite.

In addition to the updated pseudorange estimates, estimates of pseudorange errors are required. The innovation term of the EKF, Δ*x̂*, can be propagated to the line of sight (LOS) of each satellite by multiplying by a geometric matrix H, consisting of LOS vectors, to generate the error estimates:

$$\Delta \hat{\rho }=H\cdot \Delta \hat{x},$$

where:

(3) $$H=\left[\begin{array}{cccc} a_{x}^{1} & a_{y}^{1} & a_{z}^{1} & -1\\ \vdots & \vdots & \vdots & \vdots \\ a_{x}^{N} & a_{y}^{N} & a_{y}^{N} & -1 \end{array}\right]$$

$a_{x}^{k}$, $a_{y}^{k}$, $a_{z}^{k}$ : LOS elements of *x*, *y*, *z* directions, respectively, to the *k*-th satellite

Now, the reference pseudorange can be calculated by summing the updated pseudorange estimate, Equation (2), and the pseudorange error estimate, Equation (3):

(4) $${\hat{\rho }}^{+}=\hat{\rho }+\Delta \hat{\rho }=\Psi (\hat{x})+H\cdot \Delta \hat{x}$$

Finally, the tracking control input is generated by subtracting the reference pseudorange from the measured pseudorange:

(5) $$\Delta {\hat{\rho }}^{+}={\hat{\rho }}^{+}-\tilde{\rho }=\Psi (\hat{x})+H\cdot \Delta \hat{x}-\tilde{\rho }$$

**Figure 6.**
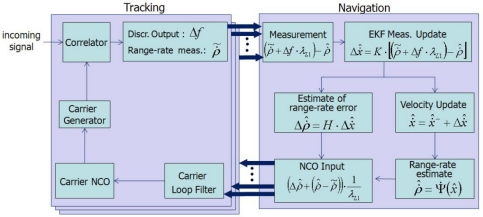
Construction of the VFLL.

Implementation of the VFLL is similar to that of the VDLL. Its workflow is shown in Figure 6. In the VFLL, the frequency discriminator output and range-rate measurements are used instead of the code discriminator output and pseudorange measurements. Velocity navigation states are used in the EKF.

VDFLL is a combined version of VDLL and VFLL. Its Kalman filter states are position and velocity elements including clock offset and clock drift error:

(6) $$\left[\begin{array}{c} \delta x_{k+1}\\ \delta y_{k+1}\\ \delta z_{k+1}\\ \delta {\dot{x}}_{k+1}\\ \delta {\dot{y}}_{k+1}\\ \delta {\dot{z}}_{k+1}\\ \delta t_{k+1}\\ \delta {\dot{t}}_{k+1} \end{array}\right]=\left[\begin{array}{cccccccc} 1 & 0 & 0 & \Delta T & 0 & 0 & 0 & 0\\ 0 & 1 & 0 & 0 & \Delta T & 0 & 0 & 0\\ 0 & 0 & 1 & 0 & 0 & \Delta T & 0 & 0\\ 0 & 0 & 0 & 1 & 0 & 0 & 0 & 0\\ 0 & 0 & 0 & 0 & 1 & 0 & 0 & 0\\ 0 & 0 & 0 & 0 & 0 & 1 & 0 & 0\\ 0 & 0 & 0 & 0 & 0 & 0 & 1 & \Delta T\\ 0 & 0 & 0 & 0 & 0 & 0 & 0 & 1 \end{array}\right]{\left[\begin{array}{c} \delta x_{k}\\ \delta y_{k}\\ \delta z_{k}\\ \delta {\dot{x}}_{k}\\ \delta {\dot{y}}_{k}\\ \delta {\dot{z}}_{k}\\ \delta t_{k}\\ \delta {\dot{t}}_{k} \end{array}\right]}_{,}$$

where

*δx*, *δy*, *δz* : linearized position states
*δẋ*, *δẏ*, *δż* : linearized velocity states
*δt* : receiver clock offset
*δ⃛* : receiver clock drift
Δ*T* : update interval

Pseudorange and range rate are used as measurements, and their model equations are:

(7) $${\hat{\rho }}_{i}=\sqrt{{\left(x_{i}-{\hat{x}}_{u}\right)}^{2}+{\left(y_{i}-{\hat{y}}_{u}\right)}^{2}+{\left(z_{i}-{\hat{z}}_{u}\right)}^{2}}+c{\hat{t}}_{u}$$

(8) $${\hat{\dot{\rho }}}_{i}=\frac{1-\frac{1}{c}\left[v_{i}-\left({\hat{\dot{x}}}_{u},{\hat{\dot{y}}}_{u},{\hat{\dot{z}}}_{u}\right)\cdot a_{i}\right]}{1+{\hat{\dot{t}}}_{u}}\cdot \lambda_{L1},$$

where:

*v_i_* : velocity vector of *i*th satellite
*a_i_*: LOS vector of user to *i*th satellite
*λ_L_*_1_: wave length of L1 carrier

The observation matrix can then be constructed as:

(9) $$\left[\begin{array}{c} \delta \rho^{1}\\ \vdots \\ \delta \rho^{N}\\ \delta {\dot{\rho }}^{1}\\ \vdots \\ \delta {\dot{\rho }}^{N} \end{array}\right]=\left[\begin{array}{cccccccc} a_{x}^{1} & a_{y}^{1} & a_{z}^{1} & 0 & 0 & 0 & -1 & 0\\ \vdots & \vdots & \vdots & \vdots & \vdots & \vdots & \vdots & \vdots \\ a_{x}^{N} & a_{y}^{N} & a_{z}^{N} & 0 & 0 & 0 & -1 & 0\\ 0 & 0 & 0 & a_{x}^{1} & a_{y}^{1} & a_{z}^{1} & 0 & -1\\ \vdots & \vdots & \vdots & \vdots & \vdots & \vdots & \vdots & \vdots \\ 0 & 0 & 0 & a_{x}^{N} & a_{y}^{N} & a_{z}^{N} & 0 & -1 \end{array}\right]\left[\begin{array}{c} \delta x\\ \delta y\\ \delta z\\ \delta \dot{x}\\ \delta \dot{y}\\ \delta \dot{z}\\ \delta t\\ \delta \dot{t} \end{array}\right]$$

**Figure 7.**
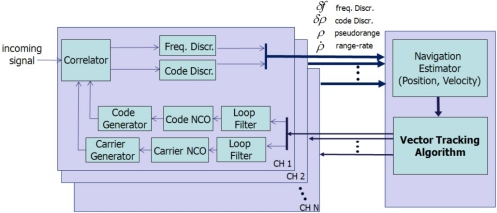
VDFLL implementation.

Figure 7 shows the VDFLL implementation combining VDLL and VFLL, as described in Figures 5 and 6, respectively:

## 4. Asynchronous PL Navigation System

**Figure 8.**
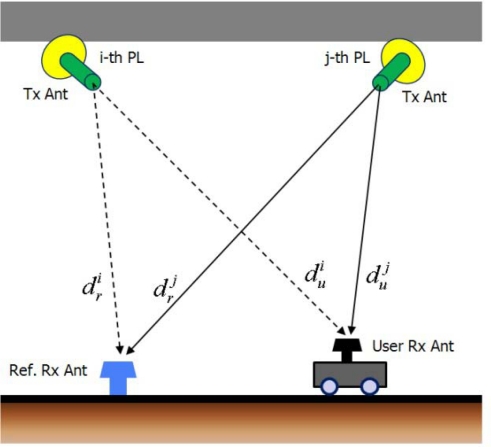
Measurement model of an asynchronous PL navigation system [19].

This section deals with the navigation algorithm for an asynchronous PL navigation system. Figure 8 shows the construction for measurement modeling. While GNSS satellites are all synchronized, asynchronous PLs are not. A reference station that transmits PL clock offset information to users is therefore required.

Pseudorange and carrier phase measurements of user and reference are modeled as follows.

Pseudorange measurement:

(10) $$\begin{array}{l} \rho_{r}^{i}=\left(R^{i}-R_{r}\right)\cdot {\hat{e}}_{r}^{i}+B_{\rho_{r}}-b^{i}+i_{r}^{i}+t_{r}^{i}+m_{\rho_{r}}^{i}+\varepsilon_{\rho_{r}}^{i}\\ \rho_{u}^{i}=\left(R^{i}-R_{u}\right)\cdot {\hat{e}}_{u}^{i}+B_{\rho_{u}}-b^{i}+i_{u}^{i}+t_{u}^{i}+m_{\rho_{u}}^{i}+\varepsilon_{\rho_{u}}^{i} \end{array}$$

where:

$\rho_{r}^{i}$, $\rho_{u}^{i}$: reference and user pseudorange of *i*th PL
*R^i^* : *i*th PL position
*R_r_*, *R_u_* : reference and user receiver positions
*B_ρ_r__*, *B_ρ_u__* : reference and user receiver clock offsets
*b^i^* : *i*th PL clock offset
$e_{r}^{i}$, $e_{u}^{i}$: reference and user LOS vectors to *i*th PL, respectively
*i, t, m, ε* : ionospheric delay, tropospheric delay, multipath delay, and thermal noise

Carrier phase measurement:

(11) $$\begin{array}{l} \varphi_{r}^{i}=\left(R^{i}-R_{r}\right)\cdot {\hat{e}}_{r}^{i}+B_{\rho_{r}}-b^{i}+i_{r}^{i}+t_{r}^{i}+m_{\rho_{r}}^{i}+\lambda \cdot N_{r}^{i}+\varepsilon_{\rho_{r}}^{i}\\ \varphi_{u}^{i}=\left(R^{i}-R_{u}\right)\cdot {\hat{e}}_{u}^{i}+B_{\rho_{u}}-b^{i}+i_{u}^{i}+t_{u}^{i}+m_{\rho_{u}}^{i}+\lambda \cdot N_{u}^{i}+\varepsilon_{\rho_{u}}^{i} \end{array}$$

where:

$\varphi_{r}^{i}$, $\varphi_{u}^{i}$: reference and user carrier phase measurement of *i*th PL
*λ* : wavelength of PL carrier (L1 = 19 cm)
*N* : ambiguity integer

To derive the navigation equation for an asynchronous PL navigation system, reference and user measurements are differenced to eliminate receiver and PL clock offset terms. Single-differenced pseudorange measurements for the receiver clock offset elimination are:

(12) $$\begin{array}{l} {\;}^{i}\nabla^{j}\rho_{r}=\rho_{r}^{i}-\rho_{r}^{j}\\ \;\;\;\;\;\;\;\;\;\;\;\;\;\;\;\;\;\;=\left(R^{i}-R_{r}\right)\cdot {\hat{e}}_{r}^{i}-\left(R^{j}-R_{r}\right)\cdot {\hat{e}}_{r}^{j}-{\;}^{i}\nabla^{j}b+{\;}^{i}\nabla^{j}t_{r}+{\;}^{i}\nabla^{j}m_{\rho_{r}}+{\;}^{i}\nabla^{j}\varepsilon_{\rho_{r}}\\ {\;}^{i}\nabla^{j}\rho_{u}=\rho_{u}^{i}-\rho_{u}^{j}\\ \;\;\;\;\;\;\;\;\;\;\;\;\;\;\;\;\;\;=\left(R^{i}-R_{u}\right)\cdot {\hat{e}}_{u}^{i}-\left(R^{j}-R_{u}\right)\cdot {\hat{e}}_{u}^{j}-{\;}^{i}\nabla^{j}b+{\;}^{i}\nabla^{j}t_{u}+{\;}^{i}\nabla^{j}m_{\rho_{u}}+{\;}^{i}\nabla^{j}\varepsilon_{\rho_{u}}, \end{array}$$

where:

*^i^*∇*^j^ρ_r_*, *^i^*∇*^j^ρ_u_* : reference and user single-differenced pseudorange between *i*th and *j*th PLs

The double-differenced measurement for satellite clock offset elimination is obtained as follows:

(13) $$\begin{array}{l} {\;}^{i}\nabla^{j}{}_{u}\Delta_{r}\rho ={\;}^{i}\nabla^{j}\rho_{u}-{\;}^{i}\nabla^{j}\rho_{r}\\ \;\;\;\;\;\;\;\;\;\;\;\;\;\;\;\;\;=\left(R^{i}-R_{u}\right)\cdot {\hat{e}}_{u}^{i}-\left(R^{j}-R_{u}\right)\cdot {\hat{e}}_{u}^{j}-\left(R^{i}-R_{r}\right)\cdot {\hat{e}}_{r}^{i}+\left(R^{j}-R_{r}\right)\cdot {\hat{e}}_{r}^{j}\\ \;\;\;\;\;\;\;\;\;\;\;\;\;\;\;\;\;\;\;\;+{\;}^{i}\nabla^{j}{}_{u}\Delta_{r}t+{\;}^{i}\nabla^{j}{}_{u}\Delta_{r}m_{\rho }+{\;}^{i}\nabla^{j}{}_{u}\Delta_{r}\varepsilon_{\rho }\;, \end{array}$$

where:

$${\;}^{i}\nabla^{j}{}_{u}\Delta_{r}(\cdot )={\;}^{i}\nabla^{j}{\left(\cdot \right)}_{u}-{\;}^{i}\nabla^{j}{\left(\cdot \right)}_{r}=\left[{\left(\cdot \right)}_{u}^{i}-{\left(\cdot \right)}_{u}^{j}\right]-\left[{\left(\cdot \right)}_{r}^{i}-{\left(\cdot \right)}_{r}^{i}\right].$$

With double-differenced pseudorange measurements containing no receiver or PL clock offset terms, the navigation equation can be constructed as:

(14) $$\begin{array}{l} \left({\hat{e}}_{u}^{j}-{\hat{e}}_{u}^{i}\right)\cdot R_{u}={\;}^{i}\nabla^{j}{}_{u}\Delta_{r}\rho -R^{i}\cdot {\hat{e}}_{u}^{i}+R^{j}\cdot {\hat{e}}_{u}^{j}+\left(R^{i}-R_{r}\right)\cdot {\hat{e}}_{r}^{i}-\left(R^{j}-R_{r}\right)\cdot {\hat{e}}_{r}^{j}+{\;}^{i}\nabla^{j}{}_{u}\Delta_{r}\varepsilon_{\rho }\\ \;\;\;\;\;\;\;\;\;\;\;\;\;\;\;\;\;\;\;\;\;\;\;\;\;\;\;\;\;\equiv z_{ij}+{\;}^{i}\nabla^{j}{}_{u}\Delta_{r}\varepsilon \end{array}$$

(15) $$\left[\begin{array}{c} {\hat{e}}_{u}^{2}-{\hat{e}}_{u}^{1}\\ {\hat{e}}_{u}^{3}-{\hat{e}}_{u}^{2}\\ \vdots \\ {\hat{e}}_{u}^{m}-{\hat{e}}_{u}^{m-1} \end{array}\right]\cdot R_{u}=\left[\begin{array}{c} z_{12}\\ z_{23}\\ \vdots \\ z_{m-1,m} \end{array}\right]+\left[\begin{array}{c} {\;}^{1}\nabla^{2}{}_{u}\Delta_{r}\varepsilon_{\rho }\\ {\;}^{2}\nabla^{3}{}_{u}\Delta_{r}\varepsilon_{\rho }\\ \vdots \\ {\;}^{m-1}\nabla^{m}{}_{u}\Delta_{r}\varepsilon_{\rho } \end{array}\right]$$

## 5. VTL Implementation in an Asynchronous PL Navigation System

As seen in Section 4, the navigation algorithm for the PL navigation system uses double-differenced measurements. Thus, satellite and receiver clock offset terms are not included in navigation states. In the VTL algorithm, estimation of pseudorange and range rate requiring the clock offset and drift terms is a necessary procedure for deducing tracking control input. The measurement model equations are repeated in Equations (16) and (17) below. It is assumed that indoor navigation occurs in an area of several square meters so that ionospheric and tropospheric delays are negligible, and multipath error was ignored for simplicity:

(16) $$\rho_{u}^{i}=\left(R^{i}-R_{u}\right)\cdot {\hat{e}}_{u}^{i}+B_{\rho_{u}}-b^{i}+\varepsilon_{\rho_{u}}^{i}$$

(17) $${\dot{\rho }}_{u}^{i}=\left({\dot{R}}^{i}-{\dot{R}}_{u}\right)\cdot {\hat{e}}_{u}^{i}+{\dot{B}}_{\rho_{u}}-{\dot{b}}^{i}+\varepsilon_{{\dot{\rho }}_{u}}^{i}$$

As these equations show, receiver and satellite clock offset and drift terms are inevitable in reconstructing the pseudorange and range rate. Therefore, the conventional construction of navigation equation described by Equations (14) and (15) is not suitable for VTL implementation.

We propose a new approach to constructing the navigation equation modified for VTL implementation. The measurement model equation is first modified below by inserting the satellite clock offset term, *b*^1^, of the reference PL. PL 1 is assumed to be the reference:

(18) $$\begin{array}{c} \rho_{u}^{1}=d_{u}^{1}+B_{u}-b^{1}+\left(b^{1}-b^{1}\right)+\varepsilon_{u}^{1}\\ \rho_{u}^{2}=d_{u}^{2}+B_{u}-b^{1}+\left(b^{1}-b^{2}\right)+\varepsilon_{u}^{2}\\ \vdots \\ \rho_{u}^{N}=d_{u}^{N}+B_{u}-b^{1}+\left(b^{1}-b^{N}\right)+\varepsilon_{u}^{N} \end{array}$$

where:

$d_{u}^{i}$: distance from user to *i*th satellite.

Now, the receiver clock offset minus the PL 1 clock offset, *B_u_* − *b*^1^, becomes the common error in the equation. It could therefore be a state variable of the navigation EKF. Each equation has the PL 1 clock offset minus the relevant PL clock offset, (*b*^1^ − *b^i^*). This term can be estimated from measurements of the reference receiver.

The single-difference pseudorange and the carrier phase measurement referenced to PL 1 in a reference receiver are:

(19) $$\begin{array}{l} {\;}^{1}\nabla^{i}\rho_{r}={\;}^{1}\nabla^{i}d_{r}-{\;}^{1}\nabla^{i}b+{\;}^{1}\nabla^{i}\varepsilon_{r,\rho }\\ {\;}^{1}\nabla^{i}\varphi_{r}={\;}^{1}\nabla^{i}d_{r}-{\;}^{1}\nabla^{i}b+{\;}^{1}\nabla^{i}N\cdot \lambda +{\;}^{1}\nabla^{i}\varepsilon_{r,\rho }, \end{array}$$

where ^1^∇*^i^b* = *b*^1^ − *b^i^*.

To estimate (*b*^1^ − *b^i^*) precisely, a Hatch filter should be applied. The Hatch filter equation for single-difference pseudorange with carrier phase measurement is:

(20) $$\begin{array}{l} \delta^{1}\nabla^{i}\rho_{r}\approx \delta^{1}\nabla^{i}\varphi_{r}\\ \to {\;}^{1}\nabla^{i}{\hat{\rho }}_{r}(k+1)=\frac{N-1}{N}\left({\;}^{1}\nabla^{i}{\hat{\rho }}_{r}(k)+\delta^{1}\nabla^{i}\varphi_{r}(k)\right)+\frac{1}{N}\delta^{1}\nabla^{i}\rho_{r}(k+1), \end{array}$$

where:

*N*: Hatch filter constant
*δ*∇*x*(*k*) = ∇*x*(*k*) − ∇*x*(*k* − 1)

Then the satellite clock offset difference, (*b*^1^ − *b^i^*), can be estimated as:

(21) $${\;}^{1}\nabla^{i}\hat{b}={\;}^{1}\nabla^{i}d_{r}-{\;}^{1}\nabla^{i}{\hat{\rho }}_{r}$$

Now, the modified navigation equation for an asynchronous PL is constructed in Equation (22). The term *B_u_* – *b*^1^ is added as a filter state variable and the satellite clock offset differenced to reference PL 1 moves to the measurement side:

(22) $$\left[\begin{array}{c} \rho_{u}^{1}{-}^{1}\nabla^{1}\hat{b}\\ \rho_{u}^{2}{-}^{1}\nabla^{2}\hat{b}\\ \vdots \\ \rho_{u}^{N}{-}^{1}\nabla^{N}\hat{b} \end{array}\right]=\left[\begin{array}{cccc} a_{x}^{1} & a_{y}^{1} & a_{z}^{1} & 1\\ a_{x}^{2} & a_{y}^{2} & a_{z}^{2} & 1\\ \vdots & \vdots & \vdots & \vdots \\ a_{x}^{N} & a_{y}^{N} & a_{z}^{N} & 1 \end{array}\right]\left[\begin{array}{c} x\\ y\\ z\\ B_{u}-b^{1} \end{array}\right]+\varepsilon$$

In this form, the receiver clock offset minus the satellite clock offset of PL 1 can be obtained as a navigation solution. Therefore, pseudorange estimation for generation of the VTL control input is possible, as shown in Equation (23). The velocity navigation equation, range-rate measurements and clock drift terms are used in the same way as in Equation (19) to derive the modified equation for the velocity navigation filter:

(23) $$\begin{array}{ll} \rho_{u}^{i} & =\left(R^{i}-R_{u}\right)\cdot {\hat{e}}_{u}^{i}+B_{\rho_{u}}-b^{i}+\varepsilon_{\rho_{u}}^{i}\\ & ≅\left(R^{i}-R_{u}\right)\cdot {\hat{e}}_{u}^{i}+\left(B_{\rho_{u}}-b^{1}\right)+{\;}^{1}\nabla^{i}\hat{b}+\varepsilon_{\rho_{u}}^{i} \end{array}$$

The final navigation equation for VDFLL in PL navigation is obtained as Equation (24), containing eight states including six position and velocity terms, the receiver clock offset minus the satellite clock offset of PL 1 and the receiver clock drift minus the satellite clock drift of PL 1:

(24) $$\left[\begin{array}{c} \rho_{u}^{1}{-}^{1}\nabla^{1}\hat{b}\\ \vdots \\ \rho_{u}^{N}{-}^{1}\nabla^{N}\hat{b}\\ {\dot{\rho }}_{u}^{1}{-}^{1}\nabla^{1}\hat{\dot{b}}\\ \vdots \\ {\dot{\rho }}_{u}^{N}{-}^{1}\nabla^{N}\hat{\dot{b}} \end{array}\right]=\left[\begin{array}{cccccccc} a_{x}^{1} & a_{y}^{1} & a_{z}^{1} & 0 & 0 & 0 & 1 & 0\\ \vdots & \vdots & \vdots & \vdots & \vdots & \vdots & \vdots & \vdots \\ a_{x}^{N} & a_{y}^{N} & a_{z}^{N} & 0 & 0 & 0 & 1 & 0\\ 0 & 0 & 0 & a_{x}^{1} & a_{y}^{1} & a_{z}^{1} & 0 & 1\\ \vdots & \vdots & \vdots & \vdots & \vdots & \vdots & \vdots & \vdots \\ 0 & 0 & 0 & a_{x}^{N} & a_{y}^{N} & a_{z}^{N} & 0 & 1 \end{array}\right]\left[\begin{array}{c} x\\ y\\ z\\ \dot{x}\\ \dot{y}\\ \dot{z}\\ B_{u}-b^{1}\\ {\dot{B}}_{u}-{\dot{b}}^{1} \end{array}\right]$$

**Figure 9.**
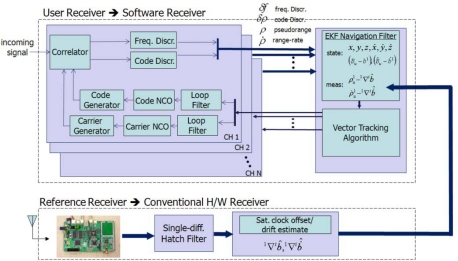
VTL implementation with a new navigation equation model.

With the new navigation equation and additional measurements, a block diagram of the VTL implementation for our asynchronous PL navigation system is shown in Figure 9. VTL was implemented in a software receiver and the reference receiver supplies satellite clock offset and drift estimates through the Hatch filter.

As an implementation issue, it is important that the user and the reference receiver should acquire measurements at the same time in order to make the terms of the left side of Equation (24). To synchronize measurement acquisition time, both receivers are set to trigger measurement acquisition at the each end of C/A code period of the reference PL 1. For GPS, one C/A code period is 1ms long equivalently converted to about 300 km. So, 1 ms resolution is valid in the use of PLs. Timing offset caused by the difference between a user to PL 1 distance and a reference to PL 1 distance is negligible in a several meters’ coverage indoor PL system. If the system would be implemented in a wider area, the timing offset and transport delay from the reference to the user should be compensated using extrapolation of clock model. In this paper, both error terms could be negligible, because this is a meter level system implemented in a small indoor area.

## 6. GPS Simulation

**Figure 10.**
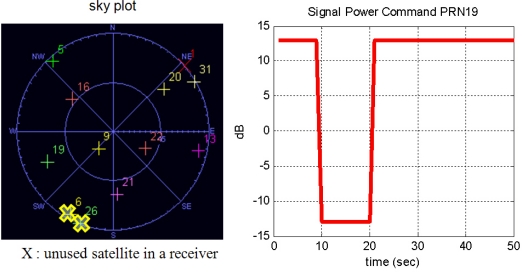
Sky plot (left) and signal power command on PRN 19 (right), (PRN 6 and PRN 26 are not used because of their low elevation angle).

To verify the performance of the implemented VTL, a simulated GPS signal generated from STR 4500 was applied [20]. The simulation scenario is a rapidly received signal power attenuation on one satellite. This is a widely used simulation method to verify the tracking performance of VTL against momentary signal blockage or attenuation. The purpose of this simulation is to see how well the VTL can handle attenuated channel tracking compared with the conventional signal tracking algorithm. Figure 10 shows the satellite constellation and the signal power command on PRN 19, the target satellite.

**Figure 11.**
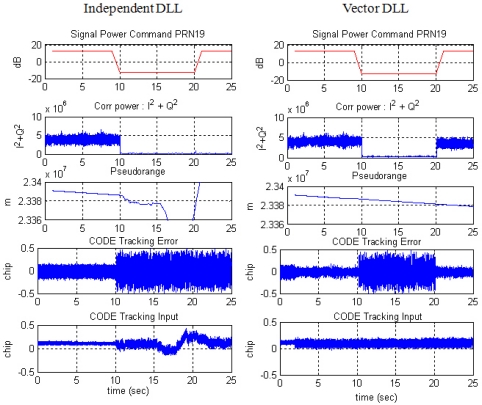
Code tracking result of PRN 19: Independent DLL (left) and Vector DLL (right).

**Figure 12.**
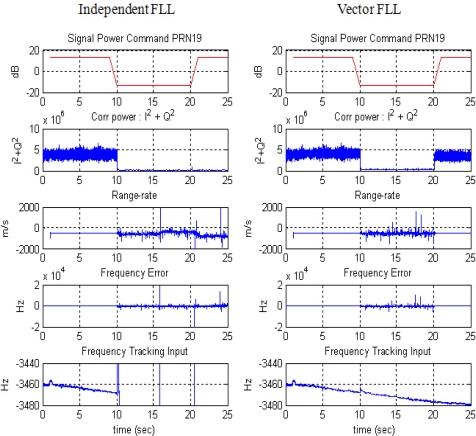
Code tracking result of PRN 19: Independent FLL (left) and Vector FLL (right).

The duration of the attenuation is 10 s, between the 10 and 20 s marks in Figure 10. Figures 11 and 12 show the code and carrier frequency tracking results for PRN 19. The first row of plots shows the simulator command setting, the second row shows the tracked signal power obtained by correlation magnitude, Σ I^2^ + Q^2^, where I and Q are in-phase and quadrature-phase accumulation values, respectively, the third row shows pseudorange or range-rate measurements, the fourth row shows discriminator output and the fifth shows tracking control input.

During the attenuation period, the correlation power level is very low, meaning that the attenuation causes a momentary blockage of the signal. For that period, neither independent DLL nor FLL could generate appropriate tracking control inputs. VDLL and VFLL, however, can continue generating control input and obtain pseudorange and range-rate measurements comparing with diverging measurements of ITLs. Verification of VTL estimation during the period can be identified by the continuous tracking after the attenuation period. As opposed to the independent tracking, VTL recovers correlation power instantly, ensuring that the implemented VTL works well and tracks the momentarily blocked signal successfully.

## 7. Experiments

### 7.1. Test Environment

**Figure 13.**
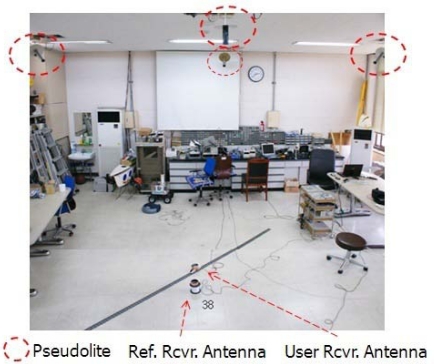
SNUGL indoor PL navigation system.

The proposed VTL algorithm was tested in an indoor asynchronous PL navigation system in the SNUGL. Navigation was based on pseudorange measurements. However, the available space in the SNUGL indoor PL navigation system, a 7 × 7 m plane and 3 m height, is not large enough for code-based navigation. We therefore applied a moving average filter to the navigation results just to see how well the VTL works and to verify VTL performance. The filtered results are not applied to the tracking loop algorithm; they are only for monitoring the navigation results. Figure 13 shows the SNUGL indoor PL navigation system and test setup.

**Figure 14.**
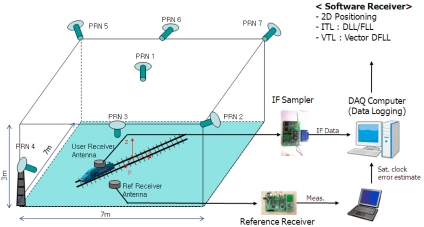
Test environment.

Figure 14 presents a more specific description of the test environment. The user receiver antenna was installed on a toy train moving along a track oriented towards the PL PRN 4. The reference receiver antenna was fixed on the floor. Because implementation of real-time VTL was not possible at the time of the experiment, the interfrequency (IF) sampled signal received by the user was stored for the postprocessing software receiver on which VTL and ITL were implemented. A commercial-grade GPS receiver based on the GP2000 chipset was used as a reference receiver and the measurements were also logged to be processed in a software receiver.

The goal of this test was to study and compare the tracking performance of VTL and ITL in the presence of the near–far problem as the user moves between the near and far zones of PL PRN 4. The tracking results of VTL and ITL, implemented on a software receiver processing stored IF sample data with logged reference receiver measurements, were therefore compared [21].

Three tests and one experimental test were performed. First, the user moved from the near to the far zone of PL PRN 4. This was the situation in which one of the PL signals was attenuated by other strong near signals. Second, the user moved to the near zone from the far area of PRN 4 and so the other far signals were to be attenuated by one strong PL signal, PRN 4. Third, the test environment was same to the first one except that the number of visible satellites is limited to three. As an experimental test, signal tracking performance of VTL with three visible satellites, the marginal number of satellites for 2-dimensional positioning, was studied.

### 7.2. Test 1: From Near to Far Zone of PRN 4

**Figure 15.**
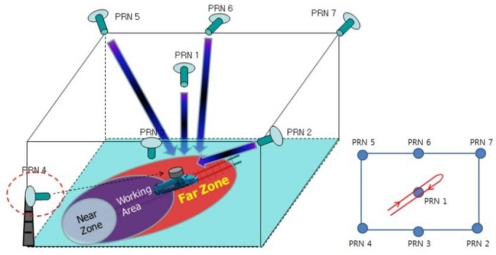
Test 1: Test setup and user trajectory.

In Test 1, the user moves from the near to the far zone of PRN 4. Figure 15 shows the test environment and the user trajectory.

**Figure 16.**
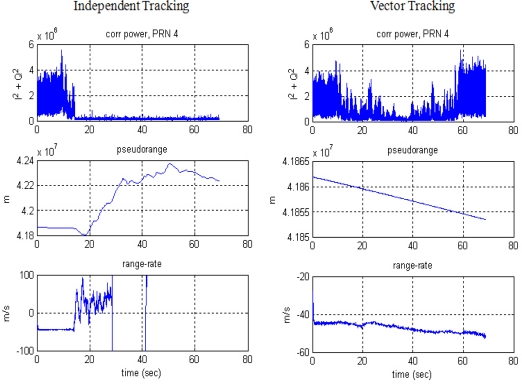
Tracking results of PRN 4: ITL (left) and VTL (right).

Figure 16 shows the tracking results for the PRN 4 signal, comparing the ITL and VTL. After about 15 s, the user has moved away from PL PRN 4 into the far zone. The correlation power of ITL starts to decrease and then the user loses its tracking lock on PRN 4. Diverging pseudorange and range-rate measurements identify the loss of the lock.

Compared with ITL, VTL tracks the signal continuously, even though the tracked signal power decreases while the user stays in the far zone. The pseudorange and range-rate measurements are estimated without diverging. When the user returns to the near zone after 60 s, correlation power was recovered without a reacquisition process and measurements are collected continuously. Thus, VTL estimation was successful and effective in tracking the signal affected by the near–far problem.

### 7.3. Test 2: From Far to Near Zone of PRN 4

**Figure 17.**
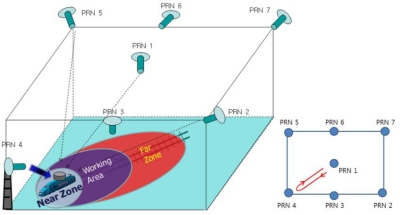
Test 2: Test setup and user trajectory.

In the second test, the user moves from the far to the near zone and then returns to the far zone of PRN 4. When the user stays in the near zone, the other PL signals suffer interference from the strong signal of the near PL signal PRN 4. Figure 17 shows the test environment and user trajectory.

**Figure 18.**
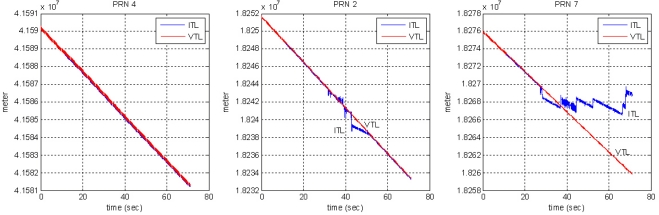
Pseudorange measurements.

**Figure 19.**
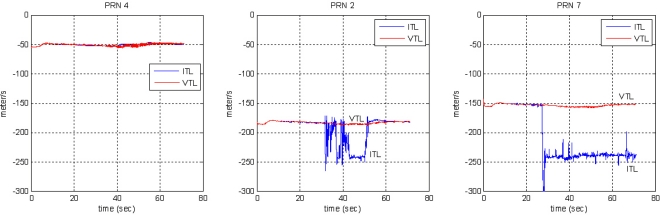
Range-rate measurements.

Figures 18 and 19 show the tracking results of the pseudorange and range rate, respectively. The arrangement of plots follows the distance from the near zone of PRN 4. As the user approaches PRN 4, the tracking performance of the far signals PRN 1, 5, 6 and 7 is disrupted in ITL, and a divergence of pseudorange measurement is observed as shown in the right plot of Figure 18 for the case of PRN 7.

**Figure 20.**
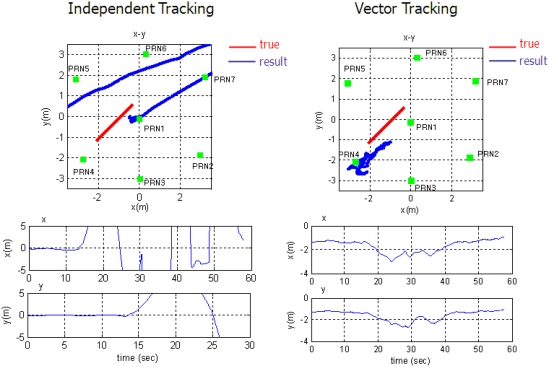
Navigation results: ITL (left) and VTL (right).

The middle plot of Figure 18 shows the tracking performance of PRN 2 signal at mid distance from the near zone. It is disturbed for a while when the user is in the near zone. As the user gets out of the zone, it resumes tracking successfully, whereas VTL continuously track the signal. The pseudorange estimation of ITL only works successfully on the near signals, PRN 3 and 4. And the tracking results of PRN 4 are shown in the left plot of Figure 18. The performance of frequency tracking and the results of range-rate measurements are similar: independent FLL could not track the far signals. The near–far problem causes frequency offset in the tracking results. Compared with ITL, VTL can track all the signals properly, even when the user stays in the near zone of PRN 4. No estimation divergence was observed in VTL for either pseudo orange or range rate. We can therefore say that VTL can track the majority of signals that are attenuated from the near–far problem for long periods Figure 20 shows the navigation results. As stated at the beginning of this section, positioning results are filtered to monitor the result. Multipath interference, caused by the small indoor environment, is assumed to be the reason for position offset from the true trajectory. Figure 20 represents only the trend of user movement. As ITL failed to track far signals when the user stayed in the near zone, the navigation results also diverge. However, the VTL results show the movement of the user, back and forth to PRN 4. This is why the coverage of the conventional PL navigation system is restricted to the area that is roughly equidistant from each PL. Our test results show that VTL can improve positioning availability and widen the coverage of a PL navigation system.

### 7.4. Test 3: From Far to Near Zone of PRN 4 with Reduced Visible Satellites

**Figure 21.**
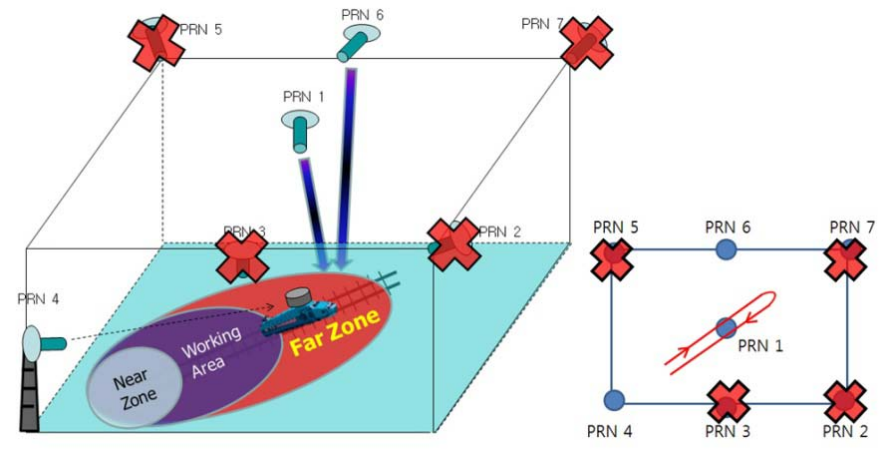
Test 2: Test setup and user trajectory.

This test was conducted to see the VTL performance in more practical situation. The number of visible satellites is restricted to three. The visible satellites are PRN 1, 4 and 6 as shown in Figure 21. The user moves from the near to the far zone of PRN 4, and returns to the near zone. In this situation, tracking availability of PRN 4 is directly connected to positioning availability.

**Figure 22.**
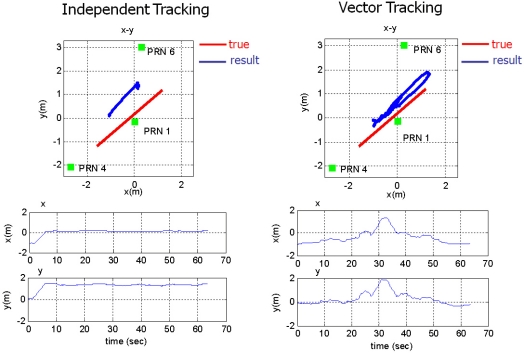
Navigation results: ITL (left) and VTL (right).

Figure 22 shows the navigation results of ITL and VTL. ITL loses tracking lock of PRN 4 as the user moves to the far zone, and then navigation filter cannot be updated. On the other hand, VTL can track the signal and perform navigation consistently. When a user moves in a urban canyon, the number of visible satellites rapidly changes. So, the consistent tracking performance of VTL could cope with momentary signal blockage.

### 7.5. Experimental Test: Tracking Performance of VTL with Marginal Number of Visible Satellites

**Figure 23.**
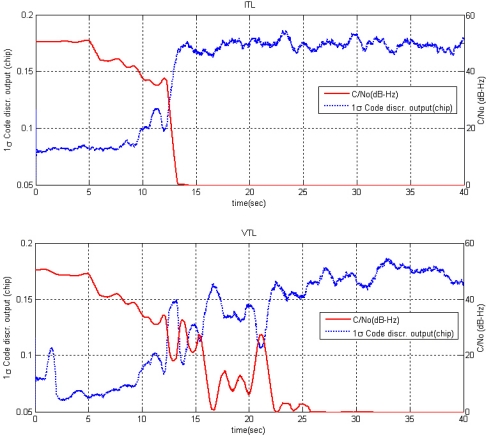
Tracking Results of PRN 4: ITL (top) and VTL (bottom).

In this test, VTL tracking performance is studied when the number of visible satellites is marginal, three for 2-dimensional positioning. The transmission power of PRN 4 decreases gradually. It is an experimental test that the steadily increasing noise was applied to the tracking channel of PRN 4 with stored IF signal to make the situation of gradual transmission power decrease in a specified PL. A code discriminator output and carrier to noise ratio (C/No) are shown in Figure 23 as tracking results of ITL and VTL, respectively. After 12 s, the code discriminator output increases rapidly and then ITL comes to lose tracking lock. It shows that ITL could keep track the signal until C/No is about 35 dB-Hz. On the other hand, VTL could maintain tracking lock until 21 seconds when C/No is about 25 dB-Hz. It shows the robust signal tracking performance of the implemented VTL.

## 8. Conclusions

We have proposed a VTL algorithm and its implementation suitable for an asynchronous PL navigation system. We presented several tests, performed using the SNUGL indoor PL navigation system, that showed that VTL has improved tracking performance in the presence of the near–far problem that is one of the major limitations in PL navigation systems. First, we proposed a modified navigation equation for asynchronous PL navigation systems applicable to VTL implementation. VDFLL in the PL system was implemented on a software receiver with a conventional hardware reference receiver. The implemented VDFLL was verified by tracking tests of a GPS simulator signal. Two kinds of field tests were conducted. In the first test, the user moved away from a specific PL, *i.e.*, the user stayed in the far zone and the far signal suffered severe interference from many other strong near signals and the conventional tracking loop could not cope. VTL, however, successfully estimated the signal and tracked it successfully. In the second test, the user moved to the near zone. We showed that even when the majority of signals are disrupted by a strong near signal, the implemented VDFLL could track them continuously. From the navigation results, even when the user moved close to a specific satellite, we verified that a PL navigation system with VTL receiver could succeed. And the third test and the experimental test showed the robust signal tracking performance of the implemented VTL in more practical situation. Thus, VTL could be a cure to the near–far problem and enhance the coverage of PL navigation systems. Recently, there have been many studies of VTL. To improve GNSS navigation availability, PL is very useful because it transmits a similar signal to the GNSS, so that the conventional GNSS user is not required to change receiver hardware. While we addressed VTL application in PL navigation systems against the near–far problem to increase navigation coverage, our tests have the deficiency that the test area was too small to do code navigation. Further tests in wider areas are necessary and tests of integrated navigation of GNSS-PL will be our future work.

## References

[1] Parkinson B, Spilker J (1996). Global Positioning System: Theory and Applications.

[2] Kanli OM Limitations of Pseudolite Systems Using Off-The-Shelf GPS Receivers.

[3] Lee T Robust GPS Receiver for Indoor Navigation System by Utilizing Long Integration Time and Data-less Pseudolite.

[4] Klein D, Parkinson B (1986). The Use of Pseudo-satellites for Improving GPS Performance. In Global Positioning System: Papers Published in NAVIGATION. Inst. Navigation.

[5] Copps EM, Geier GJ, Fidler WC, Grundy PA (1984). Optimal Processing of GPS Sensors. In Global Positioning System: Papers Published in NAVIGATION. J. Inst. Navigation.

[6] Sennott J, Senffner D The Use of Satellite Constellation Geometry and A Priori Motion Constraints for Prevention of Cycle Slips in a GPS Signal Processor.

[7] Spilker JJ (1995). Vector Delay Lock Loop Processing of Radiolocation Transmitter Signals.

[8] Benson D Interference Benefits of a Vector Delay Lock Loop (VDLL) GPS Receiver.

[9] Kaplan ED (2005). Understanding GPS : Principles and Applications.

[10] Lashely M, Bevly DM Analysis of Discriminator Based Vector Tracking Algorithms.

[11] Pany T, Eissfeller B Use of a Vector Delay Lock Loop Receiver for GNSS Signal Power Analysis in Bad Signal Conditions.

[12] Lashley M, Bevly DM A Comparison of the Performance of a Non-Coherent Deeply Integrated Navigation Algorithm and a Tightly Coupled Navigation Algorithm.

[13] Kiesel S, Held MM, Trommer GF Realization of a Deeply Coupled GPS/INS Navigation System Based on INS-VDLL Integration.

[14] Petovello MG, Lachapelle G Comparison of Vector-Based Software Receiver Implementations with Application to Ultra-Tight GPS/INS Integration.

[15] Kiesel S, Ascher C, Gramm D, Trommer GF GNSS Receiver with Vector Based FLL-Assisted PLL Carrier Tracking Loop.

[16] Zhodzishsky M, Yudanov S, Veitsel V, Ashjaee J Co-OP Tracking for Carrier Phase.

[17] Lashley M, Bevly DM Vector Delay/Frequency Lock Loop Implementation and Analysis.

[18] Brown RG, Hwang PYC (1997). Introduction to Random Signals and Applied Kalman Filtering.

[19] Yun D, Jun H, Kee C Robust and Practical Indoor Navigation System and Its Application to Automatic Control of Miniature Vehicle.

[20] (2002). STR4500 GPS/SBAS Simulator with Simplex Software User Manual.

[21] So H, Jun H, Kee C A New Three Dimensional Signal Search and Acquisiton Algorithm Based on Cross-correlation Sequence with 0.1 Seconds’ Signal Receiving Time.

